# Posterior Midline Activation during Symptom Provocation in Acute Stress Disorder: An fMRI Study

**DOI:** 10.3389/fpsyt.2014.00049

**Published:** 2014-05-08

**Authors:** Jan C. Cwik, Gudrun Sartory, Benjamin Schürholt, Helge Knuppertz, Rüdiger J. Seitz

**Affiliations:** ^1^Department of Clinical Psychology and Psychotherapy, University of Wuppertal, Wuppertal, Germany; ^2^Mental Health Research and Treatment Center, Department of Clinical Psychology and Psychotherapy, University of Bochum, Bochum, Germany; ^3^Department of Neurology, University of Düsseldorf, Düsseldorf, Germany

**Keywords:** acute stress disorder, trauma, symptom provocation, fMRI, precuneus

## Abstract

Functional imaging studies of patients with post-traumatic stress disorder showed wide-spread activation of midline cortical areas during symptom provocation, i.e., exposure to trauma-related cues. The present study aimed at investigating neural activation during exposure to trauma-related pictures in patients with acute stress disorder (ASD) shortly after the traumatic event. Nineteen ASD patients and 19 healthy control participants were presented with individualized pictures of the traumatic event and emotionally neutral control pictures during the acquisition of whole-brain data with a 3-T fMRI scanner. Compared to the control group and to control pictures, ASD patients showed significant activation in midline cortical areas in response to trauma-related pictures including precuneus, cuneus, postcentral gyrus, and pre-supplementary motor area. The results suggest that the trauma-related pictures evoke emotionally salient self-referential processing in ASD patients.

## Introduction

Acute stress disorder (ASD) is a trauma- and stress-related disorder following a traumatic event. The diagnostic criteria are intrusive re-experiencing of the trauma, autonomic reactivity in response to and avoidance of trauma-related cues, dissociation, mood deterioration, and elevated arousal that last for a minimum of 3 days and at the longest 1 month after the trauma (1). Trauma victims showing similar symptoms 1 month after the traumatic event are given the diagnosis of post-traumatic stress disorder (PTSD). A number of neuroimaging studies and subsequent meta-analyses (2–5) have been carried out on the neuronal circuit underlying PTSD. There is, however, a paucity of studies in ASD.

The key neuronal structures that were proposed to underlie PTSD are a hyperresponsive amygdala and a hyporeactive anterior cingulate cortex (ACC). The amygdala has been shown to be essential for fear conditioning (6) and to exhibit hyperresponsivity to trauma-related cues in PTSD patients [e.g., (7)]. The ACC has been associated with emotional regulation (8) and found to show diminished activity in trauma victims when confronted with trauma-related stimuli [e.g., (9)]. Additionally, a deficient hippocampal function was proposed to prevent the reassessment of the traumatic event [e.g., (10)]. Neuroimaging studies used PET or fMRI to investigate the neuronal response to symptom provocation, i.e., the presentation of trauma reminders such as pictures, sounds, or script-driven imagery of the traumatic event in chronic PTSD patients [e.g., (7, 11, 12)]. Results were, however, only partly convergent and recent meta-analyses revealed additionally activated cortical areas which were not components of the proposed circuit (4, 5). The reasons for the inconsistent results are due to a number of reasons such as methodological differences between studies, viz. region-of-interest (ROI) versus whole-brain analysis, but also to the clinical heterogeneity of the PTSD patients (13) and their variable chronic comorbid disorders with a high proportion of the patients suffering from depression and from substance-related disorders.

It is noteworthy that the unique clinical features of PTSD namely, re-experiencing and flashbacks, were not accounted for by the proposed neuronal circuit. The meta-analyses by Hayes et al. (4) and Sartory et al. (5) attempted to address this issue. Hayes et al. (4) analyzed the results of 12 symptom provocation studies and employed activation likelihood estimation, the results of which are based on peak locations of significant activation clusters. Sartory et al. (5) entered 19 neuroimaging studies of symptom provocation in patients and healthy trauma-exposed participants into effect size-signed differential mapping (ES-SDM) (14), a further development that combines peak coordinates and statistical parametric maps, and represents effect-sizes (15). Trauma patients as compared to controls with regard to their response to trauma-related stimuli as well as compared to neutral stimuli revealed increased activation in midline structures including the retrosplenial cortex, precuneus, ACC, in addition to the amygdala. The posterior midline structures have been implicated in self-referential processing and salient autobiographical memory independently of sensory modality (16–21). Moreover, retrosplenial cortex has been shown to be essential for forming associations between multiple sensory stimuli in rodents (22) and for learning contextual associations and priming in humans (23, 24). Accordingly, it was hypothesized that the trauma-related stimuli elicited autobiographically relevant memories in the trauma patients. This is supported by the finding that priming has an important function with regard to the intrusive re-experiencing of the traumatic event (25).

Compared to the large number of studies in chronic PTSD patients, there are only few in recent trauma victims. Among them is an early PET study of bank officials who had experienced an armed robbery and were shown the security video (26). Compared to a control video, the traumatic stimulation elicited increased activity in visual cortex, posterior gyrus cinguli, and left orbitofrontal cortex, and decreased activity in, among others, Broca’s area and angular gyrus. Although the results suggested altered activity in brain regions associated with cognition and affect, in the absence of control participants, it remained unclear whether the trauma victims had merely reacted to the emotional salience of the video or whether self-referential processes were involved. The patients were also not assessed for ASD.

In the study by Osuch et al. (27), the majority of the patients endorsed symptoms in each of the ASD symptom clusters. Motor-vehicle collision survivors and non-traumatized controls were exposed to trauma- and neutral-scripts. Unlike controls, ASD participants showed decreased activity in the amygdala and hippocampus and increased activity in medial prefrontal cortex in response to the trauma scripts. Decreased activity of the left amygdala was related to subsequent clinical improvement and the authors concluded that the activation pattern during exposure to trauma reminders underlay adaptive processes. As the authors carried out hypotheses-driven ROI analyses which were confined to amygdalar and frontal brain areas rather than a whole-brain analysis, possible activation in posterior brain regions may have escaped their notice.

So far, it is unclear whether, similar to PTSD patients, ASD patients exhibit activation of posterior midline areas implicated in self-referential processing and salient autobiographical memory. It is conceivable that the latter are the result of long-term consolidation of the trauma memory and that ASD is characterized by a frontal network, i.e., a hyperactive amygdala and hyporeactive ACC.

In the present study, personalized trauma-related pictures were used as in previous studies in PTSD patients (9, 11, 28–31). Similar to specific phobics [e.g., (32)] and unlike controls, ASD patients were found to show heart-rate acceleration to personalized trauma pictures (33) indicative of a fear response. The extent of the heart-rate acceleration was also found to be related to severity of intrusions and the risk of developing PTSD in ASD patients (34).

The aim of the present study was to investigate the neural basis of symptom provocation in ASD patients. Patients with a confirmed diagnosis of ASD and healthy control participants took part. Whole-brain analyses were carried out. We expected trauma victims to show increased activation in amygdala and decreased activation of medial prefrontal areas.

## Materials and Methods

### Participants

Nineteen participants with ASD [15 F, 4 M; mean age 35.5 years (SD = 13.7)] and 19 healthy control participants who had not experienced a traumatic event [10 F, 9 M; mean age 29.6 years (SD = 12.0)] took part in the study. All participants were right-handed according to the Edinburgh Handedness Inventory (35). Another 10 trauma victims were excluded because of premature termination of the scanning procedure (3), metallic implants (2), medication (2), equipment malfunction (2), and exaggerated movement (1). The traumatic event had occurred on average 16.95 days (SD = 6.55; range 9–31 days) prior to the fMRI procedure. Participants were recruited via the local police department and accident and emergency departments of hospitals, among other sources. The following traumatic incidences were reported: break-in/robbery (11), traffic accident (5), and violent threat/assault (3). Comorbid disorders among ASD participants at the time of assessment were: depression (4), panic disorder/agoraphobia (3), specific phobia (1), general anxiety disorder (1), and dysthymia (1). Control participants underwent a clinical assessment and the presence of a disorder was an exclusion criterion in this group. The study was approved by the Ethics Committees of the Universities of Wuppertal and Düsseldorf. All participants gave their written informed consent before entering the study and received a small remuneration to cover travel expenses. After the study, trauma-focused cognitive behavior therapy was offered to all patients.

### Stimuli

**Figure 1:**
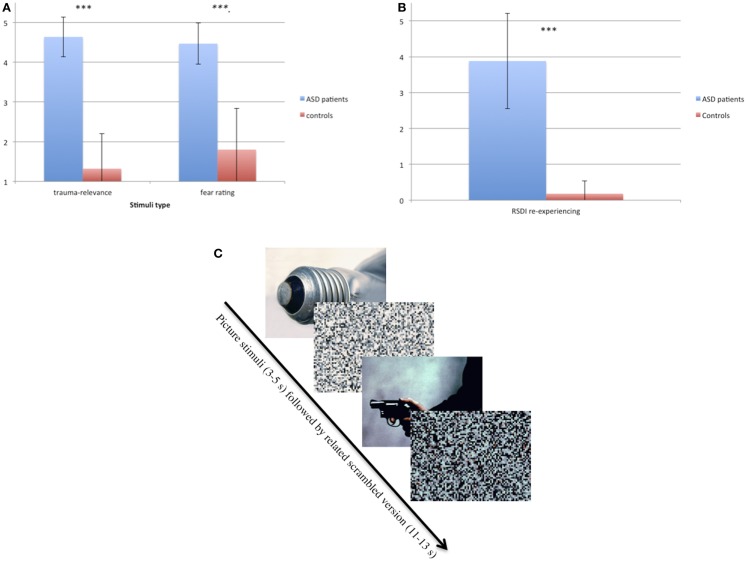
**(A)** Ratings of trauma relevance (1–5) and fear-inducing ratings (1–5) of trauma-relevant pictures in ASD and control participants. ***p < 0.001. **(B)** Ratings of re-experiencing measured with RSDI (0–6) in ASD and control participants immediately after the symptom provocation procedure in the scanner. ***p < 0.001. **(C)** Example of the two types of pictures (neutral and trauma-related) taken from IAPS (36) and their scrambled version.

At the first telephone contact, ASD participants were asked to describe the images occurring to them during intrusions. Based on this information, a set of 20 personalized trauma-relevant and 20 emotionally neutral pictures matched in color and general content (e.g., faces) were chosen for the experiment. Pictures were taken from the international affective picture system (IAPS) (36), the internet or, if available, from press or police reports covering the event. ASD participants were asked to rate the 40 pictures according to how much they reminded them of the trauma and how much fear they induced, each on a five-point scale (SuperLab Version 2.0; Cedrus Corporation, CA, USA) (see Figures 1A,B). Fifteen trauma pictures with the highest and 15 of the neutral pictures with the lowest rating of trauma relevance were chosen as stimulus material for the respective ASD participant. Each control participant was matched with one of the ASD patients with regard to the presented pictures. Additionally, scrambled versions of the pictures were produced by adjusting them to 600 × 800 pixels and scrambling them into squares of 10 × 10 pixels (see Figure 1C). In the scanner, pictures were presented for 3–5 s, followed by the respective scrambled version for 11–13 s (Figure 1C).

### Imaging method

MRI data scanning was performed at the Department of Neurology, University of Düsseldorf, on a Siemens Magnetom TRIO 3-T MRI scanner. Echoplanar $T_{2}^{*}$-weighted imaging (EPI) was obtained whole-brain in 136 images with 44 slices [repetition time (TR) 4 s, echo time (TE) 40 ms, flip angle 90°, matrix 128 × 128, field of view (FOV) 192 mm × 192 mm, pixel size 1.5 mm × 1.5 mm, 3 mm slice thickness, interleaved-even). No pictures were presented during the acquisition of three initial “dummy” volumes. High-resolution *T*_1_-weighted structural images were acquired for each participant using a magnetization-prepared gradient echo sequence in 192 slices with a voxel size of 1 mm × 1 mm × 1 mm (TR = 2.3 s, TE = 2.98 ms, flip angle = 90°, FOV = 256 mm × 256 mm, matrix = 256 × 256) for localization and coregistration of the functional data. Stimuli onsets were recorded using RTEwin (RTEwin^®^ software; Version 1.81, MH GmbH, Erftstadt)^1^ .

Image processing and statistical analysis were performed with Statistical Parametric Mapping (SPM8, Wellcome Department of Neurology, London, UK)^2^. Data were realigned and unwarped (using fourth degree B-Spline and motion parameters) and slice time corrected. After coregistration to the structural images, EPI images were spatially normalized to Montreal Neurological Institute (MNI) standard space with a voxel size of 3 mm × 3 mm × 3 mm and smoothed with a 9-mm full-width-at-half-maximum (FWHM) Gaussian kernel. A high-pass filtering with a cut-off at 128 s was used to minimize the impact of serial autocorrelations in the fMRI time series. Parameter estimation was corrected for temporal autocorrelations using a first-order autoregressive model and motion. Using convolving stick functions with the canonical hemodynamic response function (HRF), and parameter estimates pertaining to the amplitude of the HRF, each experimental condition (*trauma* >* neutral*, *neutral* >* trauma*) was calculated. As recommended by Francati et al. (37), neutral pictures were used as intrapersonal and healthy control group as interpersonal baseline.

### Clinical measures and questionnaires

*Acute Stress Disorder Interview* [ASDI; (38); German translation: (39)] was used to assess diagnostic ASD criteria. *Anxiety Disorder Interview Schedule* [ADIS-IV; (40); German version: Mini-DIPS; (41)] was given to both groups. In addition to assessing diagnostic criteria of anxiety, affective, and somatoform disorders, there are also screening questions as to psychotic and substance-related disorders in the German version of the ADIS. *Impact of Event Scale* [IES-R; (42); German version: (43)]: this self-rated questionnaire assesses trauma severity in terms of avoidance, intrusiveness, and hyperarousal. *Beck Depression Inventory* [BDI-II; (44); German version: (45)] and the *State-Trait Anxiety Inventory* [STAI; (46); German version, (47)] were used for the assessment of depression and anxiety, respectively. *Responses to Script-Driven Imagery Scale* [RSDI; (48); German version: (49)] is a brief self-report scale of PTSD symptoms evoked by imagery during the presentation of the trauma script. For the present study, RSDI was adjusted to symptom provocation by trauma-relevant pictures. Immediately after the scanning procedure, participants were asked to rate the extent to which re-experiencing, avoidance, and dissociation symptoms had occurred on a scale from “0 = not at all” to “6 = very intense.”

### Procedure

Acute stress disorder participants were informed about the trauma study by the cooperating institutions. Either participants themselves or the institutions established contact. In the first telephone contact, participants were asked about the images occurring to them during intrusions. Based on the participants’ report, the set of 40 pictures was chosen. Within 1 week after the telephone contact, participants underwent the assessment (clinical structured interviews and questionnaires) and rated the picture set. According to this rating, the final 30 pictures were selected such that the pictures were the most trauma-relevant for each trauma subject. Another week later, the fMRI scan was carried out. Participants were in supine position in the scanner while the 30 pictures were shown in pseudo-randomized order (Presentation^®^ software; Version 14.9)^3^ , i.e., the same order of trauma-relevant and neutral pictures was maintained and was presented to the ASD and control participants. Each picture was projected onto a mirror mounted on the head-coil above the participant’s head. Participants were instructed not to move and to view all pictures attentively. A passive viewing task was chosen to ensure that the resulting neural activation pattern is similar to that of intrusions which are thought to occur during passive confrontation with trauma-related cues. Participants were given a button to press in case of indisposition in the scanner. Immediately after the scan, participants were asked to fill in the RSDI.

### Statistical analysis

Hypotheses were tested as planned contrasts in a random-effects model in which linear combinations of model parameters were evaluated using *t*-statistics, focusing on comparisons between traumatic versus neutral pictures. Linear contrasts were carried out to test within- and between-group differences with regard to location and intensity of the BOLD response during the picture presentation, relative to the baseline BOLD response. Functional maps of the activated voxels were constructed by comparing the signal intensity observed during the picture presentation relative to the scrambled version for each participant on a voxel-by-voxel basis. To balance the risk of Type I and Type II errors, significant clusters had to have (a) *k* > = 15 voxels; (b) *p*-value < 0.05 (FWE-corrected) in between-group comparisons and a *p*-value < 0.01 (FWE-corrected) in within-group comparisons; and (c) one or more voxels with FDR < 0.01.

The loci of observed responses were characterized by the voxel exhibiting the maximum effect size within each cluster in terms of the MNI coordinate system. The WFU Pickatlas (Version 3.0.4, Wake Forest University, School of Medicine, NC, USA) was used to localize clusters and determine average *t*-values of clusters. Within each group, a fixed-effects model was generated to examine differences between trauma and neutral pictures.

Clinical data were compared between groups using two-tailed *t*-tests. Correlation analyses were conducted to test associations of the participants’ years of education with the eigenvariate of each significant cluster of the between-group comparison as well as the within-group comparison. The statistical modeling and analysis was carried out using R for Mac OSX [Version 3.0.1; (50)]. The statistical threshold of significance for results was set at *p* < 0.05.

## Results

### Demographic and clinical variables

Demographic and clinical data are displayed in Table 1. Patients had an age range of 20–63 and controls of 19–58 years. There was no group difference with regard to age but to years of education and clinical variables (Table 1). The correlation analyses between years of education and the eigenvariate of each significant cluster of the between-group comparison as well as the within-group comparison showed no significant results (between *r* = 0.077, *p* = 0.755 and *r* = 0.183, *p* = 0.454). ASD patients rated the trauma-related pictures as reminding them strongly of their trauma and being more fear-inducing (*p* < 0.001) than the controls. The RSDI ratings revealed that the procedure evoked ASD symptoms in patients. In contrast, there were no group differences with regard to the neutral pictures.

**Table 1:** **Demographic and clinical characteristics of patients with acute stress disorder (ASD) and controls**.

	ASD		Controls		*t*-Test/χ^2^-test statistics	*p*-Value
	*N* = 19		*N* = 19			
	Mean	SD	Mean	SD		
Age (years)	35.47	13.67	29.63	11.98	*t*(36) = 1.40	0.170
Sex, F/M	15/4		10/9		χ^2^(1, *N* = 38) = 2.92	0.087
Education (years)	10.47	0.96	12.68	0.75	*t*(36) = −7.89	<0.001***
ASDI (severity of symptoms) (0–20)	14.74	2.31	–			
BDI-II (0–63)	19.32	13.58	2.63	2.91	*t*(36) = 5.237	<0.001***
IES-R intrusion (0–35)	25.42	8.44	–			
IES-R avoidance (0–40)	22.74	12.11	–			
IES-R hyperarousal (0–35)	24.68	8.39	–			
STAI-state (20–80)	48.32	9.59	30.74	3.03	*t*(36) = 7.62	<0.001***
STAI-trait (20–80)	43.63	12.49	34.95	6.04	*t*(36) = 2.73	0.010*
**TRAUMA-RELEVANT PICTURES**						
Relevance rating (1–5)	4.64	0.50	1.32	0.88	*t*(36) = 14.31	<0.001***
Fear rating (1–5)	4.47	0.52	1.80	1.04	*t*(36) = 10.06	<0.001***
**NEUTRAL PICTURES**						
Relevance rating (1–5)	1.05	0.15	1.00	0.00	*t*(36) = 1.46	0.154
Fear rating (1–5)	1.06	0.11	1.06	0.15	*t*(36) = 0.01	0.994
RSDI re-experiencing (0–6)	3.88	1.33	0.18	0.36	*t*(36) = 11.70	<0.001***
RSDI avoidance (0–6)	2.95	1.86	0.02	0.08	*t*(36) = 6.87	<0.001***
RSDI dissociation (0–6)	2.50	1.92	0.01	0.06	*t*(36) = 5.64	<0.001***

ASDI, Acute Stress Disorder Interview; BDI-II, Beck Depression Inventory (revised); IES-R, Impact of Event Scale (revised); STAI, State-Trait Anxiety Inventory; RSDI, Response to Script-Driven Imagery Scale. *p < 0.05; ***p < 0.001.

### fMRI

#### Group differences

**Figure 2:**
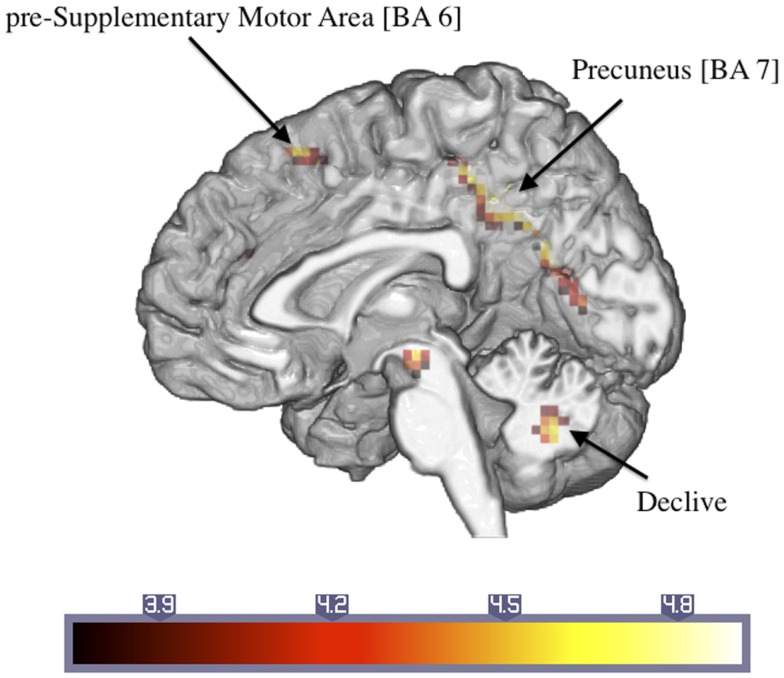
**Significantly activated areas in ASD patients compared to controls with regard to the response to the trauma-related versus neutral pictures (number in brackets indicate Brodmann areas)**.

Table 2 shows the significantly greater activations in the ASD subjects as compared to the control subjects for the trauma-specific fMRI signal changes. Significant major clusters were observed close to the midline in left precuneus and cuneus (BA = 7, 19) as well as right superior frontal gyrus (BA = 6) and the cerebellar declive. No regions of significant hypoactivation were found (Figure 2).

**Table 2:** **Comparison between ASD patients and controls with regard to their response to trauma-related compared to neutral pictures**.

ASD patients > controls (trauma-related > neutral pictures)											
Cluster-level		Cluster breakdown						Peak-level			
*k*_E_	Mean *t*-value (SD; *p*_FWE_)	Label	*k*_E_	Mean *t*-value (SD)	BA	*k*_E_	Mean *t*-value (SD)	Peak MNI coordinates			*t* (*p*_FDR_)
367	4.26 (0.66; 0.000)	**Precuneus**	**218**	**4.26 (0.65)**	7	149	4.26 (0.67)	**−6**	**−76**	**52**	**6.42 (0.002)**
		Cuneus	29	3.80 (0.31)	19	22	3.88 (0.27)				
		Postcentral G.	22	3.81 (0.33)							
80	3.90 (0.45; 0.031)	**pre-SMA/SMA**	**56**	**3.98 (0.46)**	6	32	3.85 (0.45)	**6**	**2**	**73**	**5.02 (0.005)**
75	3.84 (0.38; 0.039)	**Declive**	**25**	**3.75 (0.36)**	18	12	3.69 (0.26)	**3**	**−82**	**−17**	**4.66 (0.007)**

*All activations are significant whole-brain-analysis effects at *p* < 0.05, FWE-corrected on cluster-level, *p* < 0.01, FDR-corrected (voxel-wise) on peak-level and a minimum of *k* = 15 contiguous voxels (135 mm^3^). Areas in bold letters refer to the coordinates of the peak-level voxels*.

*k_E_, number of voxels; BA, Brodmann-area; (pre-)SMA, (pre-)supplementary motor area; G., gyrus*.

#### ASD patients: trauma-related versus neutral pictures

**Figure 3:**
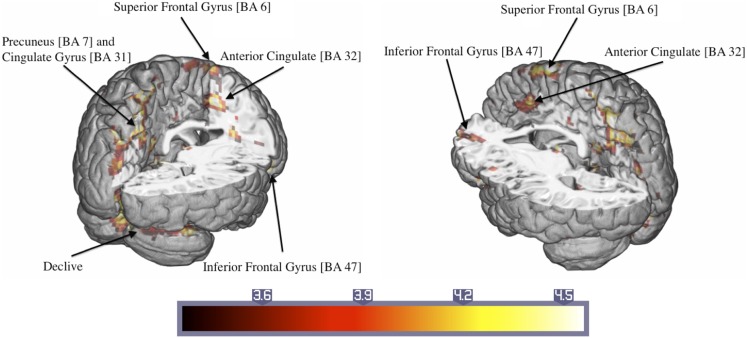
**Significant activations of the response to trauma-related compared to neutral pictures in ASD patients (numbers in brackets indicate Brodmann areas)**.

Significant activations were observed in the left precuneus (BA = 7) and posterior cingulate cortex (BA = 31) and the right superior frontal gyrus (BA = 6) (Table 3). Additionally, there were large activated areas bilaterally in the right inferior frontal gyrus (BA = 47) extending into the adjacent superior temporal gyrus as well as in the right cerebellar declive (Figure 3). No regions of significant hypoactivation were found.

**Table 3:** **ASD patients: comparison of neural activation to trauma-related as compared to neutral pictures**.

ASD patients: trauma-related > neutral pictures											
Cluster-level		Cluster breakdown						Peak-level			
*k*_E_	Mean *t*-value (SD; *p*_FWE_)	Label	*k*_E_	Mean *t*-value (SD)	BA	*k*_E_	Mean *t*-value (SD)	Peak MNI coordinates			*t*(*p*_FDR_)
787	4.02 (0.54; 0.000)	**Precuneus**	**387**	**4.05 (0.51)**	7	233	4.12 (0.54)	**−6**	**−70**	**61**	**6.12 (0.002)**
		Post. cingulate G.	46	3.78 (0.41)	31	23	3.80 (0.38)				
		Cuneus	34	3.58 (0.21)	19	23	3.55 (0.20)				
		Postcentral G.	25	3.92 (0.40)							
		Post cingulate	16	3.70 (0.27)							
388	3.96 (0.43; 0.000)	**Inf. frontal G**.	**203**	**4.05 (0.46)**	47	58	4.21 (0.53)	**−33**	**20**	**−8**	**5.48 (0.004)**
		Sup. temporal G.	82	3.79 (0.33)	38	22	3.84 (0.34)				
		Insula	38	3.94 (0.39)	13	18	4.02 (0.47)				
193	3.77 (0.33; 002)	**Inf. frontal G**.	**147**	**3.78 (0.34)**	47	38	3.79 (0.33)	**36**	**23**	**−14**	**4.71 (0.006)**
		Sup. temporal G.	29	3.81 (0.32)	38	10	4.10 (0.27)				
385	3.84 (0.41; 0.000)	Mid. cingulate G.	124	3.87 (0.39)	32	67	3.78 (0.33)				
		**Sup. frontal G**.	**114**	**3.88 (0.45)**	6	62	3.88 (0.42)	**9**	**5**	**73**	**5.13 (0.004)**
		Med. frontal G.	26	3.56 (0.18)							
		Ant. cingulate	21	3.47 (0.11)							
776	3.88 (0.35; 0.000)	**Declive**	**363**	**3.94 (0.34)**				**6**	**−85**	**−20**	**4.69 (0.006)**
		Pyramis	40	3.72 (0.29)							
		Tuber	32	3.69 (0.33)							
		Culmen	23	3.66 (0.23)							
		Declive (vermis)	20	3.91 (0.26)							

*All activations are significant whole-brain-analysis effects significant at *p* < 0.01, FWE-corrected on cluster-level, *p* < 0.01, FDR-corrected (voxel-wise) on peak-level and a minimum of *k* = 15 contiguous voxels (135 mm^3^). Areas in bold letters refer to the coordinates of the peak-level voxels*.

*k_E_, number of voxels; BA, Brodmann-area; Sup., superior; Mid., middle; Med., medial; Inf., inferior; Post, posterior; Ant., anterior; G., gyrus; L., lobule*.

#### Controls: trauma-related versus neutral pictures

**Figure 4:**
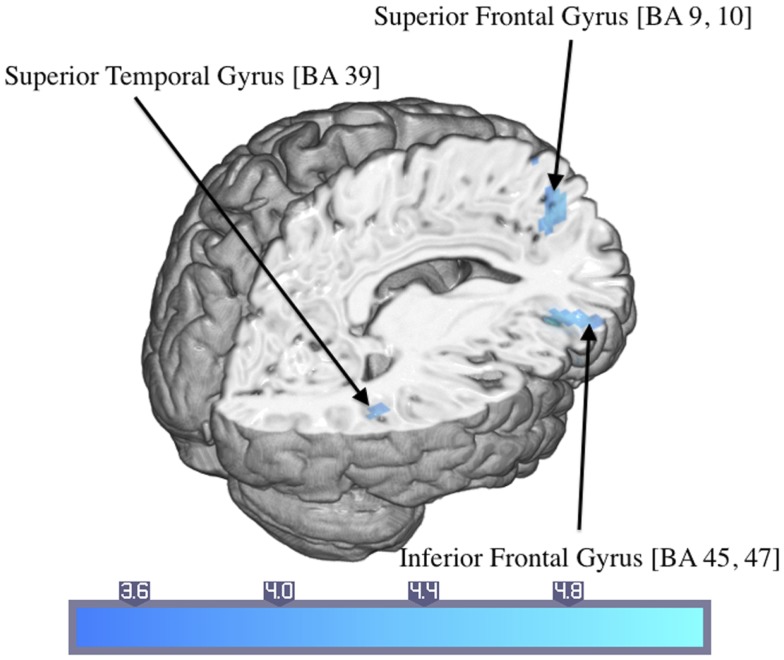
**Significant activations of the response to trauma-related compared to neutral pictures in the control group (numbers in brackets indicate Brodmann areas)**.

Control participants showed activated areas in the right inferior frontal and superior temporal gyrus as well as in the left superior frontal gyrus in response to trauma-related compared to neutral pictures (Table 4; Figure 4).

**Table 4:** **Control participants: comparison of response to trauma-related compared to neutral pictures**.

Controls: trauma-related > neutral pictures											
Cluster-level		Cluster breakdown						Peak-level			
*k*_E_	Mean *t*-value (SD; *p*_FWE_)	Label	*k*_E_	Mean *t*-value (SD)	BA	*k*_E_	Mean *t*-value (SD)	Peak MNI coordinates			*t*(*p*_FDR_)
372	4.40 (0.77; 0.000)	**Inf. frontal G**.	**289**	**4.43 (0.74)**	47	52	4.36 (0.67)	**30**	**11**	**−11**	**7.23 (0.000)**
		Insula	17	4.75 (0.92)	13	17	4.75 (0.92)				
					45	16	4.31 (0.76)				
127	3.76 (0.29; 0.007)	**Sup. frontal G**.	**90**	**3.78 (0.29)**	9	33	3.78 (0.27)	**−9**	**47**	**16**	**4.76 (0.006)**
					10	24	3.82 (0.30)				
106	3.77 (0.28; 0.007)	**Sup. temporal G**.	**49**	**3.82 (0.29)**	39	11	3.60 (0.14)	**45**	**−49**	**22**	**4.54 (0.008)**
		Inf. parietal L.	28	3.87 (0.29)							
		Supramarginal G.	16	3.59 (0.15)							

*All activations are whole-brain-analysis effects significant at *p* < 0.01, FWE-corrected on cluster-level, *p* < 0.01, FDR-corrected (voxel-wise) on peak-level and a minimum of *k* = 15 contiguous voxels (135 mm^3^). Areas in bold letters refer to the peak-level voxels*.

*k_E_, number of voxels; BA, Brodmann-area; Sup., superior; Inf., inferior; G., gyrus; L., lobule*.

## Discussion

Acute stress disorder patients rated the trauma-relevant pictures as being strongly reminiscent of their traumatic event and reported considerable more symptoms than the control participants during the procedure. However, the results of the study did not confirm the initial hypothesis of a hyperactive amygdala and hyporesponsive ACC. Instead, the ASD patients showed greater activation of midline posterior cortex including precuneus, cuneus, and posterior cingulate cortex together with greater activation in dorsal superior cortex and declive of the cerebellum. Both groups showed increased activation of inferior and medial frontal gyrus as well as superior temporal gyrus and insula in response to trauma-related as compared to control pictures. Only controls showed significant activation in left superior frontal gyrus (BA = 9, 10). ASD patients thus showed a similar pattern of activation as PTSD patients (5) with regard to posterior midline cortex.

Research in healthy samples has repeatedly shown precuneus to be a core structure of networks underlying autobiographic meaning and emotional salience processing (51–57). For instance, Lee et al. (55) observed higher neural activation in precuneus during recollection of prior negative affective events. Levine et al. (58) found episodic autobiographical remembering compared to personal semantic, general episodic memory, or semantic knowledge to elicit increased neural activation in posterior midline cortex including precuneus. Bluhm et al. (59) compared a self-referential processing condition with a general facts condition with the former showing greater neuronal responses at midline precuneus in both healthy participants and PTSD patients. Finally, Sajonz et al. (56) reported an increased BOLD response in this area to self-referential pictures. Reviewing the literature, Cavanna and Trimble (60) concluded that precuneus was involved in diverse functions among them, visual–spatial imagery, episodic memory retrieval, and self-processing operations such as first-person perspective-taking.

In the present study, the large activated cluster containing precuneus also comprised cuneus, area 31 of retrosplenial cortex and the medial aspect of the superior parietal lobule. Underscoring the integrative function of precuneus, connectivity studies revealed this area to be part of a network linking it to cuneus, lingual gyrus, middle frontal gyrus, and supplementary motor area (53, 55, 61) as well as sensorimotor areas and cerebellum in healthy (62) and traumatized samples (4). This network has been shown to be activated specifically during the recollection of autobiographical memory. For instance, Addis et al. (51) found specific autobiographic memory retrieval to be associated with increased activation of left precuneus, left superior parietal lobule, and right cuneus whereas general memory retrieval was associated with activation of the right inferior temporal gyrus, right medial frontal cortex, and left thalamus. Thus, based on these findings of the functional significance of the activation of posterior midline cortex in healthy samples, the present results may suggest that compared to controls, ASD patients showed greater self-involvement and autobiographical memory retrieval during the presentation of the trauma-related pictures. This conclusion is also confirmed by the relevance ratings given to the trauma-related pictures by ASD patients and their extent of symptom provocation during the scanning procedure but will need to be confirmed by direct measurement of the conjectured process.

Posterior cingulate gyrus together with a large network of other areas has also been identified as the default mode network activated during resting state (57). Lanius et al. (63) showed that increased connectivity with the right amygdala was predictive of PTSD symptoms 3 months post-trauma. Based on these findings, Daniels et al. (64, 65) proposed long-term structural changes to the default mode network in PTSD which would in turn incur deficits in cognitive function (57). The latter have, however, not been convincingly demonstrated in ASD or PTSD [e.g., (66)].

Compared to neutral pictures and control participants, ASD patients showed increased activation in midline right superior frontal gyrus comprising the pre-supplementary motor area and the adjacent supplementary motor area (pre-SMA/SMA, BA = 6) to trauma-related pictures. In line with studies of motor imagery (67–69), SMA has been found to be functionally connected to precuneus (53, 61, 70, 71), whereas pre-SMA is connected to dorsolateral prefrontal cortex (72). In addition to the well-known executive motor aspects (73), SMA was shown to be involved in sensory and working memory processes in healthy and traumatized participants (74–78) as well as motor inhibition (79–82). Pissiota et al. (83) observed increased neural activation in SMA during symptom provocation in a traumatized sample and suggested that this activation pattern represented a functional network supporting emotionally determined motor preparation. Alternatively, it may contain motor aspects of the memory necessary for the preparation of fight or flight. In their meta-analysis of neural activation during symptom provocation in PTSD, Hayes et al. (4) also reported significantly increased activation in SMA during unpleasant stimuli and concluded that its function was preparation to respond. Pre-SMA was found to be involved in the selection and preparation of motion (82, 84–86) but also, as shown in a meta-analysis of empathic processing, in the self-referencing of action (87). The increased pre-SMA/SMA activation of ASD patients in the present study could therefore be the result of re-experiencing motion or the preparation of a motor reaction to the trauma-related pictures.

Activation of cerebellum has also been reported previously in the context of emotional processing. In a meta-analysis of imaging studies of emotional face processing, Fusar-Poli et al. (88) found neural activation in cerebellum across all emotional conditions. The researchers interpreted the finding in terms of the arousal-related connection of cerebellum with the reticular system and with cortical association areas subserving cognitive processing of emotions. Driessen et al. (89) also found cerebellar activation during confrontation with traumatic versus unpleasant but non-traumatic key words while Osuch et al. (90) reported increased regional blood flow in cerebellar regions to be positively correlated with flashback intensity in PTSD. Presenting trauma-related pictures to earthquake victims, Yang et al. (30) found bilaterally increased activation in visual association cortex (BA = 18) and cerebellum. Along with other regions associated with autobiographic memory, cerebellum could be involved in visual re-experiencing of emotionally arousing pictures.

Activation of right inferior frontal gyrus as well as superior temporal gyrus and insula was observed to trauma-related pictures in both ASD patients and controls. Previous studies reported increased activation of inferior frontal cortex to be related to attention (91) and working memory (92, 93). Activation of insular cortex has been found to be associated with negative emotions, e.g., disgust (94, 95) and other negatively valenced reactions (96–98). The observed activation pattern could therefore be seen as a result of negative emotions such as disgust to the trauma-related pictures in both groups.

Comparing the results of the meta-analysis by Sartory et al. (5) of symptom provocation in chronic PTSD to those of the present ASD patients shows increased neural activation in precuneus in both groups and, to a lesser degree in ASD patients, retrosplenial cortex suggesting that the trauma-related pictures are associated with emotional autobiographical meaning. However, the present ASD group also showed activation of pre-SMA and cerebellar areas not evident in the chronic PTSD patients. Furthermore, the control group of the meta-analysis showed increased activation of sensory associative areas in response to the trauma-related cues which was not the case in the present control group. One of the reasons for this discrepancy could be due to controls having undergone the traumatic event without developing PTSD in the meta-analysis whereas they had not undergone such an event in the present study. Further inconsistencies among results were the increased activation of amygdala and gyrus angularis in PTSD patients not found in the present ASD patients. The discrepancy could be due to the difference in sample size between the meta-analysis and the present study or that a significant BOLD response in amygdala was only found in ROI analyses rather than the present whole-brain analysis. A direct comparison between ASD and PTSD patients would permit drawing firmer conclusions as to the activation of brain areas during the development of the disorder.

Among the limitations of this study is the lack of an additional control group that has undergone a traumatic event without subsequent ASD symptoms. Some of the emotionally driven attentional responses may be accounted for by having recently experienced a respective event. Furthermore, we compared the neural activation of ASD patients with the neural activation of control participants during the presentation of a picture set that was trauma-relevant and therefore emotionally meaningful for the ASD patient but not for controls. Future studies should include emotionally relevant pictures for controls, e.g., of life-event stress for the comparison with trauma-relevant pictures of ASD patients. Francati et al. (37) proposed including healthy or trauma-exposed controls as an interpersonal baseline in neuroimaging studies in addition to neutral stimuli as an intrapersonal baseline. We decided to include healthy controls because it appeared to be the first step in assessing characteristics of the disorder. It is, in any case, noteworthy that the present neuroimaging results are similar to those of the previous meta-analysis of PTSD patients compared to trauma-exposed controls (5).

Even though the number of 19 participants in each group approaches the recommended sample size for fMRI studies (99), a larger sample size would have increased the power of the statistical analyses. Furthermore, the incorporation of autonomic measures such as heart-rate would be useful to confirm the distressing quality of the trauma pictures in ASD patients. In addition, future studies could also include negatively valenced pictures to assess whether or not trauma patients respond generally more strongly to unpleasant stimuli or whether their reactivity is confined to personalized trauma reminders. Also of interest could be the comparison of trauma-related pictures with script-driven imagery of the trauma to investigate whether these symptom provocation methods bring about a different neural activation pattern (e.g., posterior versus limbic activation).

Finally, ASD patients of the present study showed a lower educational level than controls which is a frequently reported result in PTSD research (100). Although there was no significant correlation between neural activation and educational level in the present study, the latter is likely to have an impact particularly if cognitive tasks are involved.

## Conflict of Interest Statement

The authors declare that the research was conducted in the absence of any commercial or financial relationships that could be construed as a potential conflict of interest.

## Footnotes

^1^http://www.mh-gmbh.de/

^2^www.fil.ion.ucl.ac.uk/spm/

^3^www.neurobs.com
